# Vaginosonography versus MRI in Pre-Treatment Evaluation of Early-Stage Cervical Cancer: An Old Tool for a New Precision Approach?

**DOI:** 10.3390/diagnostics12122904

**Published:** 2022-11-22

**Authors:** Ailyn M. Vidal Urbinati, Ida Pino, Anna D. Iacobone, Davide Radice, Giulia Azzalini, Maria E. Guerrieri, Eleonora P. Preti, Silvia Martella, Dorella Franchi

**Affiliations:** 1 Preventive Gynecology Unit, European Institute of Oncology IRCCS, 20141 Milan, Italy; 2 Department of Biomedical Sciences, University of Sassari, 07100 Sassari, Italy; 3 Division of Epidemiology and Biostatistics, European Institute of Oncology IRCCS, 20141 Milan, Italy; 4 Obstetrics and Gynecology Specialization School, University of Udine, 33100 Udine, Italy

**Keywords:** vaginosonography, transvaginal ultrasound, cervical cancer, MRI and gynecological oncology diagnosis

## Abstract

This study aims to analyze the sensitivity of vaginosonography (VGS) and magnetic resonance imaging (MRI) in the preoperative local evaluation of early-stage cervical cancers and to assess their accuracy in the detection of tumors, size of the lesions and stromal invasion by comparing them with the final histopathology report. This single-center study included 56 consecutive patients with cervical cancer who underwent VGS and MRI from November 2012 to January 2021. VGS significantly overestimated the lesion size by 2.7 mm (*p* = 0.002), and MRI underestimated it by 1.9 mm (*p* = 0.11). Both MRI and VGS had a good concordance with the pathology report (Cohen’s kappa of 0.73 and 0.81, respectively). However, MRI had a false-negative rate (38.1%) that was greater than VGS (0%) in cases of cervical tumor size <2 cm. We found a good concordance between histology and VGS in the stromal infiltration assessment, with 89% sensitivity (95% CI 0.44–0.83) and 89% specificity (95% CI 0.52–0.86). VGS is a simple, inexpensive, widely available, and fast execution method that can complement ultrasound in particular cases and show a good correlation with MRI in the assessment of tumor dimensions, with a better performance in detecting small tumors (<2 cm).

## 1. Introduction

Cervical cancer is one of the leading causes of morbidity and mortality in women, representing the second most common cancer in developing countries and the fourth worldwide [1].

Although a pelvic examination continues to be the first approach to detecting cervical cancer, the 2018 FIGO classification has updated the cancer staging, highlighting the central role of imaging. In fact, imaging techniques allow a better definition of the tumor size, parametrial invasion, extension to the pelvic wall or adjacent organs, and lymph node involvement [2].

Many diagnostic methods are used in clinical practice; their use depends on availability, cost, and the expertise of clinicians and radiologists. Although magnetic resonance imaging (MRI) is considered the main technique to assess tumor size and parametrial extension [3,4,5,6], transvaginal ultrasound (TVUS) or transrectal ultrasound (TRUS) can provide comparable information (especially if performed by expert examiners) while being more widely available, faster, and cheaper [4,7,8,9,10]. Moreover, the sonographic appearance of cervical lesions seems to predict the histopathological subtype: hypoechogenicity is associated with squamous cell carcinoma in 73% of cases, while isoechogenicity suggests an adenocarcinoma in 68% of women [11]. The role of imaging in assessing vaginal invasion is less clear, as several false-negative and false-positive results have been reported for large tumors that spread to the upper vagina [2,8]. Several sonographic and anatomical factors may reduce the performance of ultrasound techniques in the detection of vaginal and distal cervix lesions, such as similar echogenicity and collapsed vaginal walls [9,11,12].

Vaginosonography (VGS) is a technique first described by Dessole et al. in 2003 [13] that combines TVUS with the vaginal instillation of saline solution or ultrasound gel. It is used to evaluate local disorders of the cervix and vagina, such as benign or malignant lesions, malformations, and infiltrating endometriosis. The acoustic window created by the passive distention of the vaginal walls allows a better analysis of the anatomical structures, making the ultrasound evaluation easier, especially in the detection of exophytic early-stage cervical cancers [13,14,15].

Our study aims to analyze the sensitivity of VGS and MRI in the preoperative local evaluation of early-stage cervical cancers and to assess their accuracy in the detection of tumor presence, size of the lesion, and stromal invasion by comparing them with the final histopathology report. 

## 2. Materials and Methods

We enrolled 81 consecutive women diagnosed with cervical cancer by clinical examination and referred to the European Institute of Oncology from November 2012 to January 2021. All patients underwent diagnostic evaluation, including a cervical biopsy or a pathological review of the original slides if the initial diagnosis was made at a different institution. All tumors were staged according to the FIGO (2018) criteria using vaginal and rectal examinations, standard chest and abdominal computed tomography (CT), TVUS or TRUS, and MRI. Twenty-five patients were excluded either because they received non-surgical treatments [12] or their FIGO stage was above IB2 [13]. Data from 56 patients were analyzed in the study. We included only the patients that underwent both VGS and MRI, whose surgical specimen was available within 30 days from the imaging evaluation, and that had early FIGO stages (≤IB2).

The results of MRI and VGS were compared with the pathology report. If the MRI scan was performed outside the European Institute of Oncology, an internal gynecological oncology radiologist reviewed the images.

All patients underwent both TVUS and VGS. The scans were performed by two experienced ultrasound examiners with 25 and 15 years of experience in ultrasound for gynecologic oncology (D.F. and A.M.V.U.), respectively. The examiners were blinded to the MRI results.

All women were examined in the lithotomy position with an empty bladder using high-end ultrasound equipment, with the frequency of the vaginal probes varying between 5.0 and 9.0 MHz. Ultrasonographic images were acquired according to a standardized time gray-scale examination technique.

The VGS procedure consists of four steps: (1) a 5.3 Fr sonohysterography catheter is inserted into the vagina by an assistant; then, (2) the probe is inserted by the operator; (3) the assistant closes the vaginal channel by narrowing the major labia; (4) approximately 60 cc of room temperature saline solution is instilled while the ultrasound is performed. 

The following parameters were evaluated by VGS and TVUS: tumor presence, maximum diameter of the tumor, parametrial and fornix infiltration, vaginal involvement, stromal invasion, anterior and posterior septum involvement, and blood flow of the lesion. The blood flow was assessed by a color Doppler score based on the intensity of the color signal with the following value ranges: (1) no flow, (2) minimal flow, (3) moderate flow, and (4) high vascular flow.

A dedicated pathologist, highly experienced in gynecologic cancers, assessed the specimens and described the size and extent of the tumor, histology type, grading, stromal invasion, minimum free thickness, and maximum deep infiltration.

Approval was obtained from the ethics committee of the European Institute of Oncology (UID 2731), and all patients signed an informed consent form.

## 3. Statistical Analysis 

Patients’ characteristics were summarized either by count and percent or mean and standard deviation (SD) for categorical and continuous variables, respectively. Methods agreement was estimated using Cohen’s kappa coefficient and Lin’s correlation. Lesion size (maximum diameter) differences between VGS and MRI with respect to histology were summarized by mean, SD, and 95% confidence intervals (95% CI) and compared using Bland–Altman and Passing–Bablok regression methods. All method comparisons considered histology as the gold standard. Cross-tabulation of the categorical variables was tested for significance using Fisher’s exact test; the mean comparison was made using the unpaired t-test. All tests were two-tailed and considered significant at the 5% level. All analyses were done using SAS 9.4 (NC, Cary) and STATA (StataCorp., College Station, Texas, USA, 2021. Stata Statistical Software: Release 17. College Station, TX: StataCorp LLC).

## 4. Results

The demographic and clinicopathological characteristics of the 56 patients included in the study and their previous treatments are reported in Table 1. The median age was 39.1 years (range 24–72), and the population was mainly Italian (92.9%). In total, 23 cancers (41%) were squamous cell carcinomas, 23 (41%) adenocarcinomas, and 10 other histotypes (18%), including 4 adenosquamous tumors, 3 clear cell variants, 2 neuroendocrine carcinomas, and 1 adenoid basal carcinoma. Invasive cervical cancer tumors were confirmed in 55 patients, while no residual tumor was found in one case. None of our patients reported any discomfort during the procedure.

Forty-two (75%) lesions were described as highly vascularized (color score 4—CS 4), 12 (21.4%) as moderately vascularized (CS 3), and 2 (3.6%) as minimally vascularized. We did not find any correlation between vascularization and histotype, but we found a statistically significant correlation between vascularization and the largest dimension of the lesion, with a mean of 26.9 mm in CS 4 and 12.4 mm in CS 3 (*p* < 0.001).

**Figure 1:**
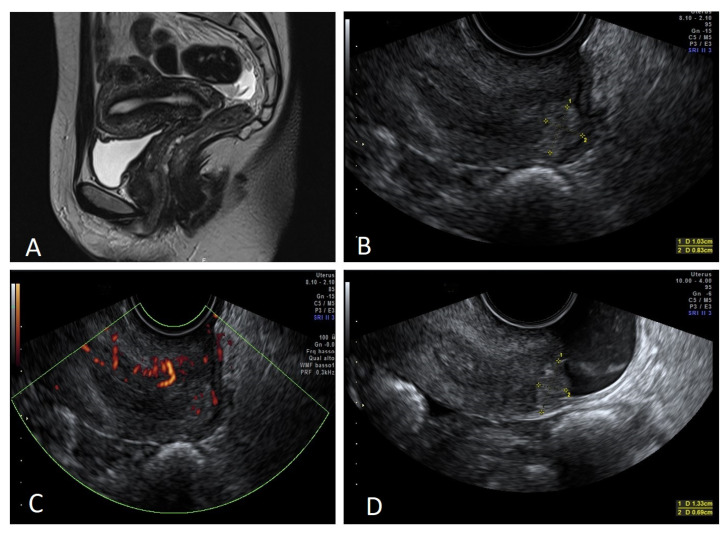
Small tumor after conization. (**A**) Not detectable disease by MRI. (**B**) Detectable disease by TVUS, measuring 10.3 × 8.3 mm. (**C**) Lesion vascularization by power Doppler. (**D**) Disease detectable by VGS, measuring 13.3 × 6.9 mm.

Out of the six patients with prior conization and residual disease, MRI detected the lesion only in three cases, while VGS detected all six cases. In Figure 1, it is possible to observe one of these cases.

**Figure 2:**
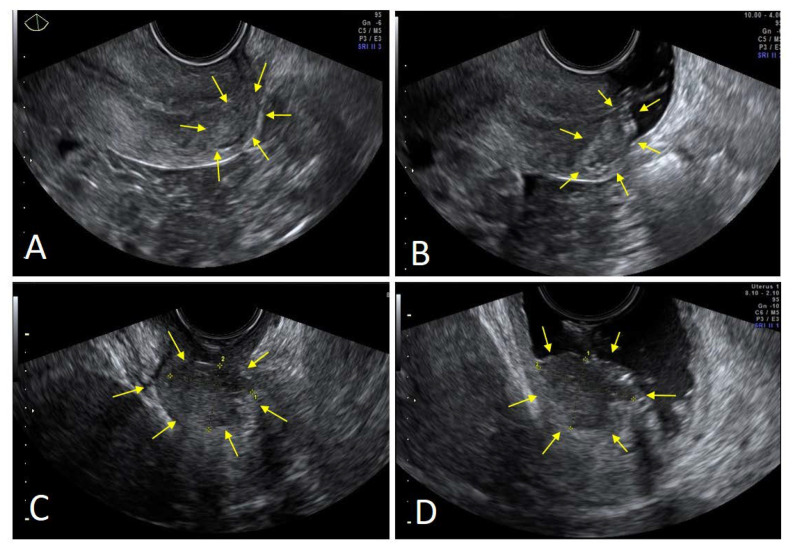
Echogenicity by TVUS and VGS. (**A**) Small adenocarcinoma (arrows) at TVUS; (**B**) the same tumor at VGS, with better definitions of the margins and more hyperechoic echogenicity (arrows). (**C**) Squamous tumors (arrows) at TVUS; (**D**) the same tumor at VGS, with better definitions of the margins and more hypoechoic echogenicity (arrows).

Squamous tumors were predominantly hypoechoic (14/23; 60.1%), while adenocarcinomas were mainly hyperechoic (16/23; 69.5%) (*p* = 0.005). Figure 2 shows an example of two echogenicities, with and without VGS.

When the stromal infiltration was analyzed, there was a good concordance between histology and VGS, with a sensitivity of 89% (95% CI 0.44–0.83), a specificity of 89% (95% CI 0.52–0.86), and a Cohen’s kappa of 0.78. When the stromal invasion was >2/3, the histological exam identified five of six (83.3%) cases with positive lymph nodes (6), while VGS predicted four cases (66.6%).

Although we did not compare the fornix infiltration to the gold standard because of the early stage of the tumors, we observed eight false-positive cases of fornix infiltration with MRI and two with VGS, with a specificity of 85.7% and 96.4% and a negative predictive value (NPV) of 88.9% and 100%, respectively.

**Figure 3:**
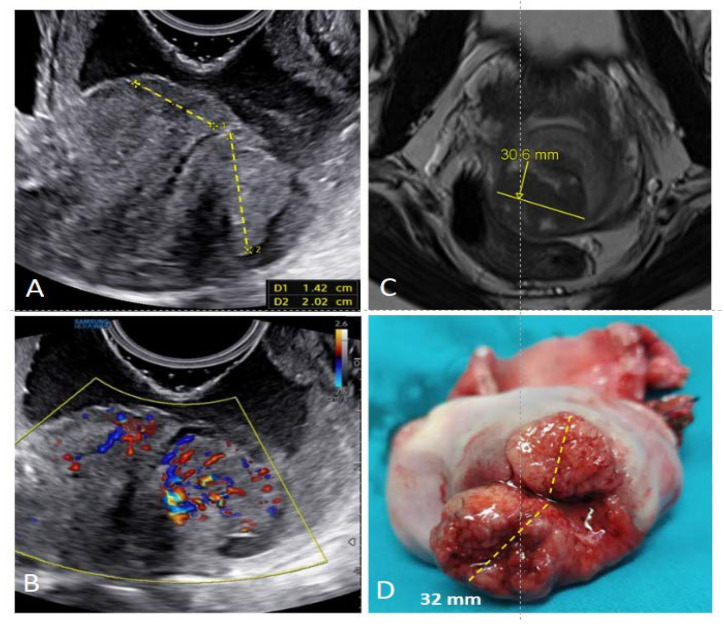
Dimensions with both techniques compared with final histology. VGS image of IB2 N+ squamous tumor (**A**,**B**), MRI (**C**), and macroscopic appearance (**D**).

VGS significantly overestimated the lesion size by 2.7 mm compared to the gold standard. On the other hand, MRI underestimated it by 1.9 mm (Table 2). Figure 3 is an example of the small difference in lesion size by the methods compared with the final specimen.

**Figure 4:**
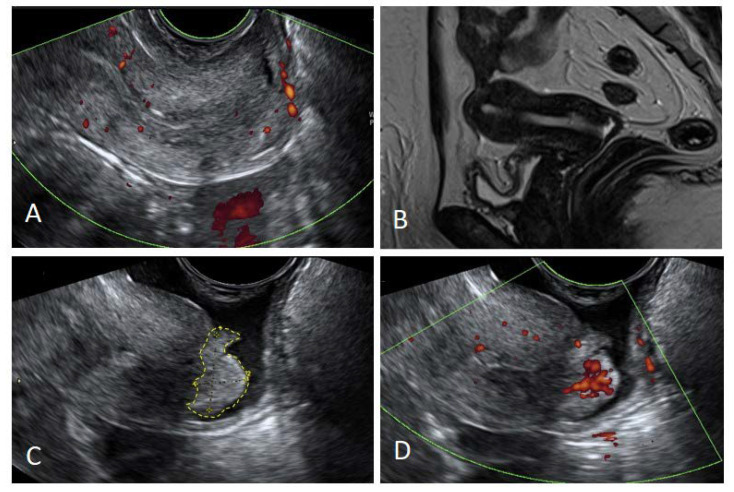
Detection of small tumors with VGS. (**A**,**B**) Small tumor not detectable at TVUS and MRI; (**C**,**D**) the same tumor detected by adding VGS and PD.

When comparing the size of the lesions, both MRI and VGS had a good concordance with the pathology report (Table 3), showing a Cohen’s kappa of 0.73 and 0.81, respectively. If the tumor was <2 cm, VGS identified 16 cases, while MRI identified 18 cases out of 21. However, in the latter group, MRI missed eight cases whose dimensions were recorded as 0 mm (Figure 2D), corresponding to a false-negative rate of 38.1% (95% CI: 18.1–61.6%). This explains the apparently higher sensitivity of MRI with respect to VGS for tumors smaller than 2 cm (Figure 4).

**Figure 5:**
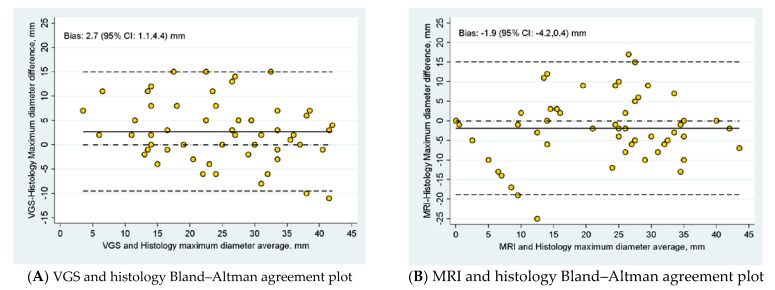

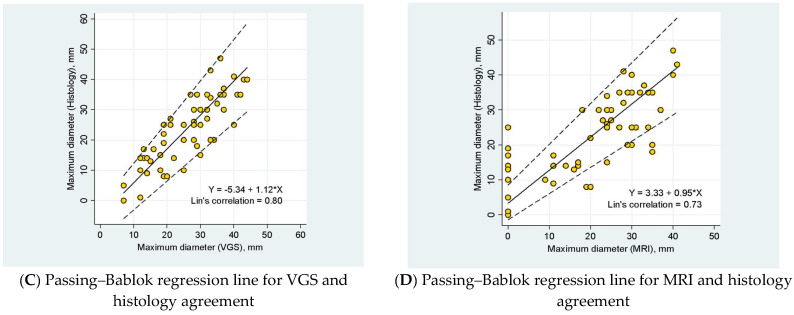
Bland–Altman plot of both methods compared with the gold standard.

Figure 5A shows the Bland–Altman plot of the maximum diameters in VGS and histopathology, while Figure 5B shows the diameter in MRI and histopathology. Visual inspection of the scatterplots suggests a good distribution of the data with both methods. The magnitude of the differences did not change with the mean of the two measurements. Overall, Figure 5C,D show no differences despite the diameter of the tumors, except for the cluster of eight tumors missed by the MRI (Figure 5B,D). Lin’s correlation was strong for VGS (0.8) and moderate for MRI (0.7).

## 5. Discussion

The role of ultrasound in the pre-treatment evaluation of cervical cancer is well established [2,16,17,18]. However, some factors hinder the ultrasound evaluation of some tumor characteristics, such as lesion margins (especially the small exophytic ones), fornix involvement, and vascular patterns [9,11,19].

To the best of our knowledge, this is the first study that investigates the accuracy of VGS in the assessment of early-stage cervical cancer. VGS is an inexpensive and well-tolerated ultrasound technique that can increase the image quality of cervical tumors. The acoustic windows created by saline solution results in a distance of the probe to the lesion, the distension of vaginal walls, and the exclusion of hindering factors, including vaginal collapse, bleeding, and/or mucus secretions.

Compared to histology (used as the gold standard), VGS and MRI assess the dimension of the lesion with similar accuracy. However, we found that VGS tended to overestimate the dimensions, whereas MRI underestimated them. Previous studies have reported that ultrasounds overestimate tumor dimensions [7,17]. Despite avoiding confounding factors, such as adjacent normal tissues, by using liquid distension in order to create an acoustic window, our data showed that the small difference between VGS and histology was statistically significant, while the difference between MRI and histology was not. Even if overestimating the tumor size could be better than underestimating it in oncological cases, it may compromise the opportunity for fertility-sparing surgery in selected patients.

The value of Lin’s concordance in the assessment of the lesion dimensions was better between VGS and histology than MRI and histology. This is because MRI did not detect some small tumors, recorded as 0 mm in our study. Indeed, when the tumor was <2 cm, MRI was not able to identify 38.1% of the tumors. Therefore, the real detection rate of MRI was 47% (10 out of 21) versus 76.1% of VGS (16 out of 21). Hence, VGS is more sensitive than MRI in identifying small lesions. Prior studies did not find that TVUS was better than MRI in the detection of lesions <2 cm [9,20]. Only one previous study showed that TRUS was superior to MRI in the identification of very small lesions (<1 cm) [7].

As our study focuses on early-stage cervical cancers, we have limited data on fornix infiltration. Nevertheless, we found that VGS was more sensitive than MRI in excluding fornix infiltration, with an NPV of 100% and 88.9%, respectively. Recently, similar results have been reported by comparing the accuracy of histology to TVUS, MRI, and clinical examination under anesthesia [21]. TVUS can be a good method to exclude but not to predict vaginal infiltration, which is often overestimated. Probably, VGS could play a role in the assessment of more advanced stages of cervical cancer with vaginal involvement.

We confirmed that different histotypes have different echogenicities, as demonstrated in a previous report [11]. Epstein et al. found that the echogenicity/histotype correlation was statistically significant, with a hypoechoic pattern in the squamous tumor cells and an isoechoic pattern in adenocarcinoma tumor cells. In our study, squamous tumor cells were predominantly hypoechoic, while the adenocarcinoma tumor cells were hyperechoic. These differences could be explained by the changes in the echogenicity of surrounding tissues due to the acoustic windows placed between the ultrasound probe and the tumor.

As observed in previous studies [7,9,11,22], almost all tumors analyzed presented moderate or intense vascularization (96.4%). The power Doppler can be useful in the assessment of the presence and borders of the tumor, especially when the echogenicity does not help. Only two adenocarcinomas were poorly vascularized. These two cases did not present any other special characteristic; both tumors were >15 mm, and the patients did not receive prior therapy nor conization; one tumor showed > 2/3 stromal invasion and the other <2/3 stromal invasion. 

Stromal infiltration is another interesting issue. Some studies reported that comparing ultrasound and MRI results with the final histology report led to high false-positive rates [8,17,18], and a prospective multicenter study found that the subjective assessment of TVUS or TRUS was better than objective measurements at predicting deep stromal invasion in patients with cervical cancer [23]. In our study, we observed a good concordance between VGS and histology (89%), with a low false-positive rate (11%). The comparison between MRI and VGS for stromal invasion was not possible because MRI data were not available in all cases.

It is a well-known matter that the incidence of lymph node metastasis in cervical cancer depends on the stage: 2% in IA2, 14–36% in IB, 38–51% in IIA, and 47% in IIB (14). Stromal infiltration is one of the independent factors identified for the risk of lymph node metastases in patients with cervical cancers, along with age, tumor size, lymph vascular space invasion, histological grade, and type [23,24]. In our series, we found six patients (11%) with lymph node metastasis; all cases were FIGO stage IB. In these patients, the stromal invasion was >2/3 in five patients out of six at the histology of cervical specimens. VGS correctly identified four out of five tumor cases with stromal invasion >2/3. The only discordant patient had a not-usual histotype of cervical cancer (adenoid basal carcinoma), with an extension of 17 mm.

## 6. Conclusions

VGS is a simple, inexpensive, widely available, and fast execution method that can complement ultrasound in particular cases. To our knowledge, this is the first study that has evaluated the diagnostic accuracy of VGS and MRI in cancer and compared it with the final histology. Our results show a good correlation between VGS and MRI in the assessment of tumor dimensions, highlighting the better performance of VGS in detecting small tumors (<2 cm) and in predicting the absence of fornix infiltration as it shows higher sensitivity.

Moreover, the acoustic windows created between the ultrasound probe and the tumor can improve the study of the echogenicity of squamous tumors and adenocarcinomas by emphasizing the hyperechogenicity of the latter. There was a very good concordance between stromal invasion predicted by VGS and histology, which is an independent factor for the risk of lymph node involvement.

However, VGS has some limitations: despite its widespread availability, it is an operator-dependent technique, and this can limit its application to centers with highly qualified examiners.

Future studies are needed to assess the use of VGS in patients with advanced tumor stages, especially the ability to correctly identify patients with vaginal spread and septal infiltration.

## Figures and Tables

**Table 1:** Patients’ characteristics and treatments, N = 56.

Characteristic	Level	Statistics *^a^*
Country of origin	Italian	49 (87.5)
	Other EU	4 (7.1)
	Non-EU	3 (5.4)
FIGO Stage	IA1	2 (3.6)
	IB1	45 (80.4)
	IB2	9 (16.1)
Pre-conization		6 (10.7)
Pre- NACHT *^b^*		4 (7.1)
Stromal invasion	<2/3	29 (51.8)
	>2/3	27 (48.2)
Echogenicity	Isoechogenic	8 (14.3)
	Hypoechogenic	21 (37.5)
	Hyperechogenic	25 (44.6)
	Mixed	2 (3.6)
Score PD	2	2 (3.6)
	3	12 (21.4)
	4	42 (75.0)
Intervention	Hysterectomy rad	43 (76.8)
	Conization	8 (14.3)
	NACHT + Hysterectomy rad	3 (5.4)
	LA + NACHT + Conization	2 (3.6)
Histology	Adenocarcinoma	23 (41.1)
	SCC	23 (41.1)
	Other *^c^*	10 (17.8)
Grading	G1	2 (3.6)
	G2	21 (37.5)
	G3	33 (58.9)
Lymph node status	pN0	50 (89.3)
	pN1	6 (10.7)
Age *^d^*, years		38.8 (8.9)
Maximum diameter of the lesion (mm) by method	Histology	23.4 (11.2)
	VGS	26.1 (9.8)
	MRI *^e^*	21.5 (12.5)
Minimum free thickness (mm) *^f^*		7.0 (4.3)
Maximum infiltration depth (mm) *^e^*		8.2 (5.9)

*^a^* Statistics are: N (%) for categorical variables, mean (SD) otherwise; SD = standard deviation; *^b^* NACHT = neoadjuvant chemotherapy; *^c^* adenosquamous N = 4, clear cell spinocellular variant N = 3, adenoid basal carcinoma N = 1, large cell neuroendocrine carcinoma N = 2; *^d^* min = 24, max = 72; *^e^* N = 49; *^f^* N = 54. VGS = vaginosonography; SCC = squamous cells carcinoma; MRI = magnetic resonance imaging.

**Table 2:** Maximum diameter (mm) methods comparison with histology (gold standard) and summary statistics.

Method	N	Mean (SD)	95% CI	*p*-Value *^a^*
Histology	56	23.4 (11.2)	(20.4, 26.4)	-
VGS	56	26.1 (9.8)	(23.5, 28.7)	-
MRI	56	21.5 (12.5)	(18.2, 24.9)	-
Bias VGS—Histology *^b^*	56	2.7 (6.3)	(1.1, 4.4)	0.002
Bias MRI—Histology *^c^*	56	−1.9 (8.5)	(−4.2, 0.4)	0.11

*^a^* Two-sided paired t-test: lower limit of agreement: *^b^* −9.5 mm, *^c^* −18.8 mm; cases under limit: *^b^* N = 2 (3.6%), *^c^* N = 1 (1.8%); upper limit of agreement: *^b^* 15.0 mm, *^c^* 15.1 mm; cases over limit: *^b^* N = 3 (5.4%), *^c^* N = 2 (3.6%).

**Table 3:** Methods comparison with the gold standard by tumor size (maximum diameter, mm).

		Size (mm) by Histology N (col %)		Cohen’s Kappa (95% CI)	*p*-Value *^a^*
Method	Size (mm)	<20 N = 21	≥20 N = 35		
VGS	<20	16 (76.2)	2 (5.7)	0.73	
	≥20	5 (23.8)	33 (94.3)	(0.54, 0.91)	0.26
MRI	<20	18 (85.7)	2 (5.7)	0.81	
	≥20	3 (14.3)	33 (94.3)	(0.65, 0.97)	0.65

*^a^* McNemar’s test.

## Data Availability

Not applicable.
